# Supplementary material for: Global Proteomics for Identifying the Alteration Pathway of Niemann–Pick Disease Type C Using Hepatic Cell Models
Source: Int J Mol Sci. 2023 Oct 27;24(21):15642. doi: 10.3390/ijms242115642 (PMC10648601; doi:10.3390/ijms242115642)
Supplement: Supplementary file 1 [file ijms-24-15642-s001.zip › Figure S1_3.2.pptx]

## Slide 1
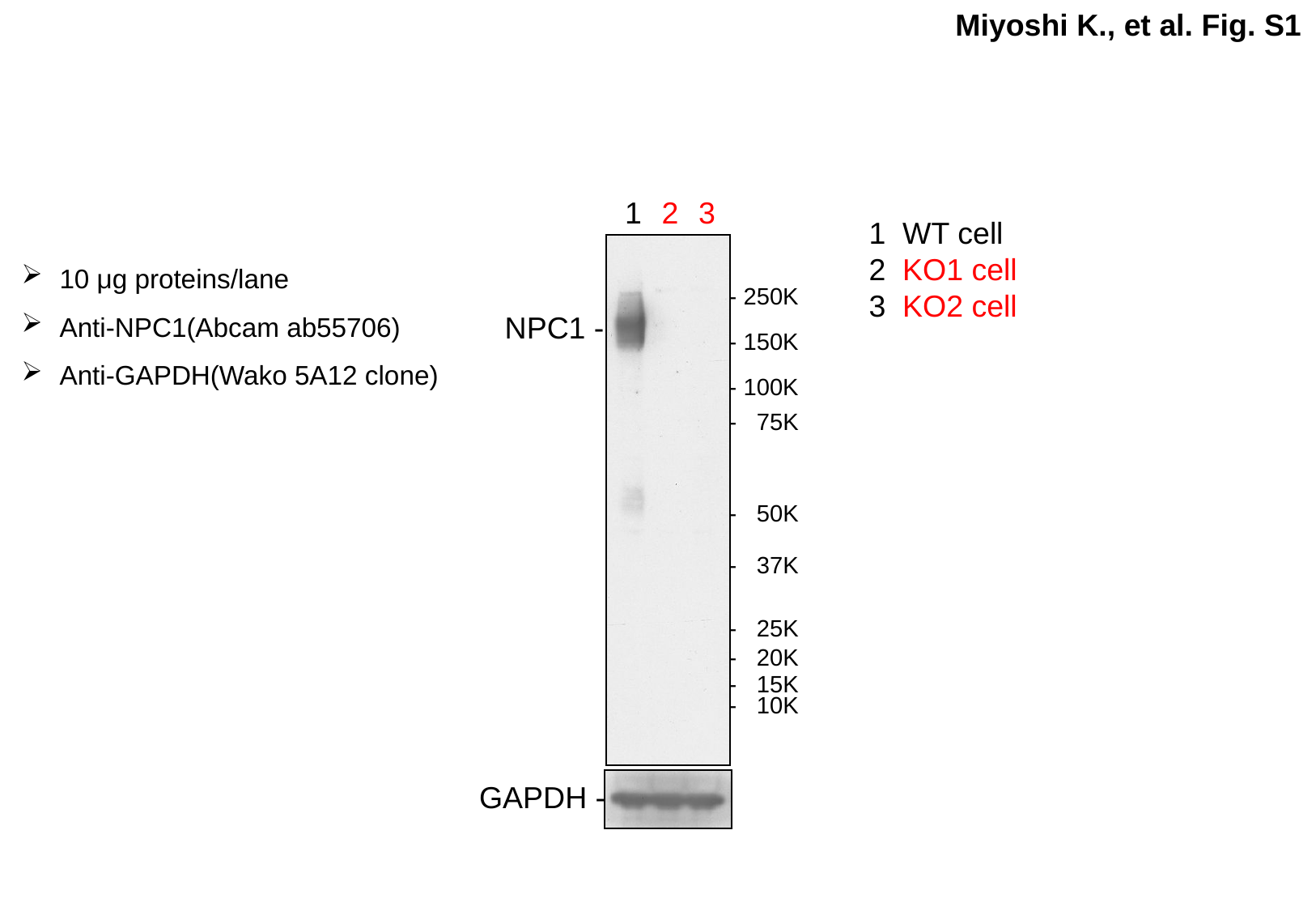

Miyoshi K., et al. Fig. S1
1
2
3
1 WT cell
2 KO1 cell
3 KO2 cell
10 μg proteins/lane
Anti-NPC1(Abcam ab55706)
Anti-GAPDH(Wako 5A12 clone)
- 250K
NPC1 -
- 150K
- 100K
- 75K
- 50K
- 37K
- 25K
- 20K
- 15K
- 10K
GAPDH -
