# Supplementary material for: Global Proteomics for Identifying the Alteration Pathway of Niemann–Pick Disease Type C Using Hepatic Cell Models
Source: Int J Mol Sci. 2023 Oct 27;24(21):15642. doi: 10.3390/ijms242115642 (PMC10648601; doi:10.3390/ijms242115642)
Supplement: Supplementary file 1 [file ijms-24-15642-s001.zip › Figure S3_3.2.pptx]

## Slide 1
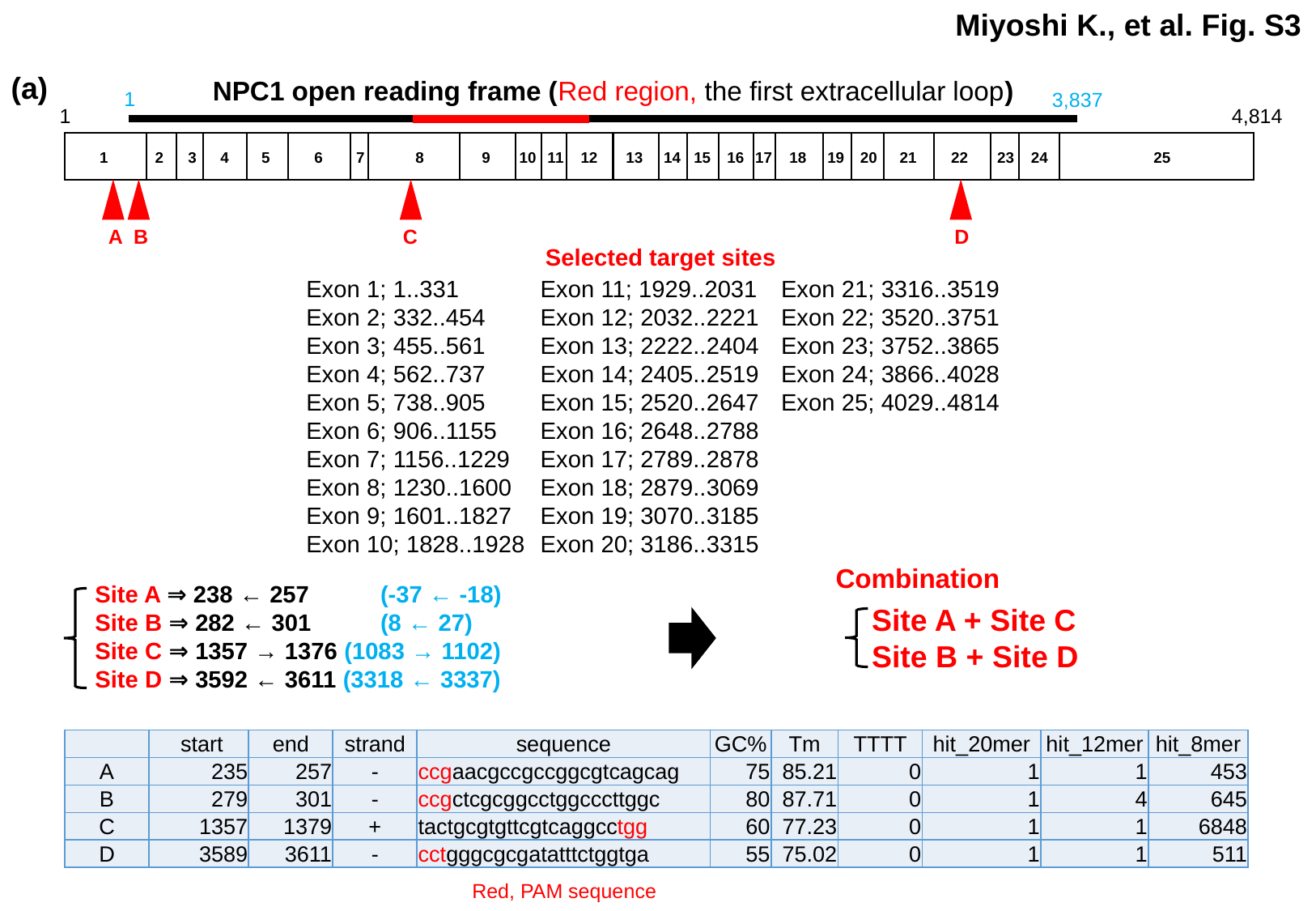

Miyoshi K., et al. Fig. S3
(a)
NPC1 open reading frame (Red region, the first extracellular loop)
1
3,837
1
4,814
1
2
3
4
5
6
7
8
9
10
11
12
13
14
15
16
17
18
19
20
21
22
23
24
25
A
B
C
D
Selected target sites
Exon 1; 1..331
Exon 2; 332..454
Exon 3; 455..561
Exon 4; 562..737
Exon 5; 738..905
Exon 6; 906..1155
Exon 7; 1156..1229
Exon 8; 1230..1600
Exon 9; 1601..1827
Exon 10; 1828..1928
Exon 11; 1929..2031
Exon 12; 2032..2221
Exon 13; 2222..2404
Exon 14; 2405..2519
Exon 15; 2520..2647
Exon 16; 2648..2788
Exon 17; 2789..2878
Exon 18; 2879..3069
Exon 19; 3070..3185
Exon 20; 3186..3315
Exon 21; 3316..3519
Exon 22; 3520..3751
Exon 23; 3752..3865
Exon 24; 3866..4028
Exon 25; 4029..4814
Combination
Site A ⇒ 238 ← 257	(-37 ← -18)
Site B ⇒ 282 ← 301	(8 ← 27)
Site C ⇒ 1357 → 1376 (1083 → 1102)
Site D ⇒ 3592 ← 3611 (3318 ← 3337)
Site A + Site C
Site B + Site D
| | start | end | strand | sequence | GC% | Tm | TTTT | hit\_20mer | hit\_12mer | hit\_8mer |
| --- | --- | --- | --- | --- | --- | --- | --- | --- | --- | --- |
| A | 235 | 257 | - | ccgaacgccgccggcgtcagcag | 75 | 85.21 | 0 | 1 | 1 | 453 |
| B | 279 | 301 | - | ccgctcgcggcctggcccttggc | 80 | 87.71 | 0 | 1 | 4 | 645 |
| C | 1357 | 1379 | + | tactgcgtgttcgtcaggcctgg | 60 | 77.23 | 0 | 1 | 1 | 6848 |
| D | 3589 | 3611 | - | cctgggcgcgatatttctggtga | 55 | 75.02 | 0 | 1 | 1 | 511 |
Red, PAM sequence

## Slide 2
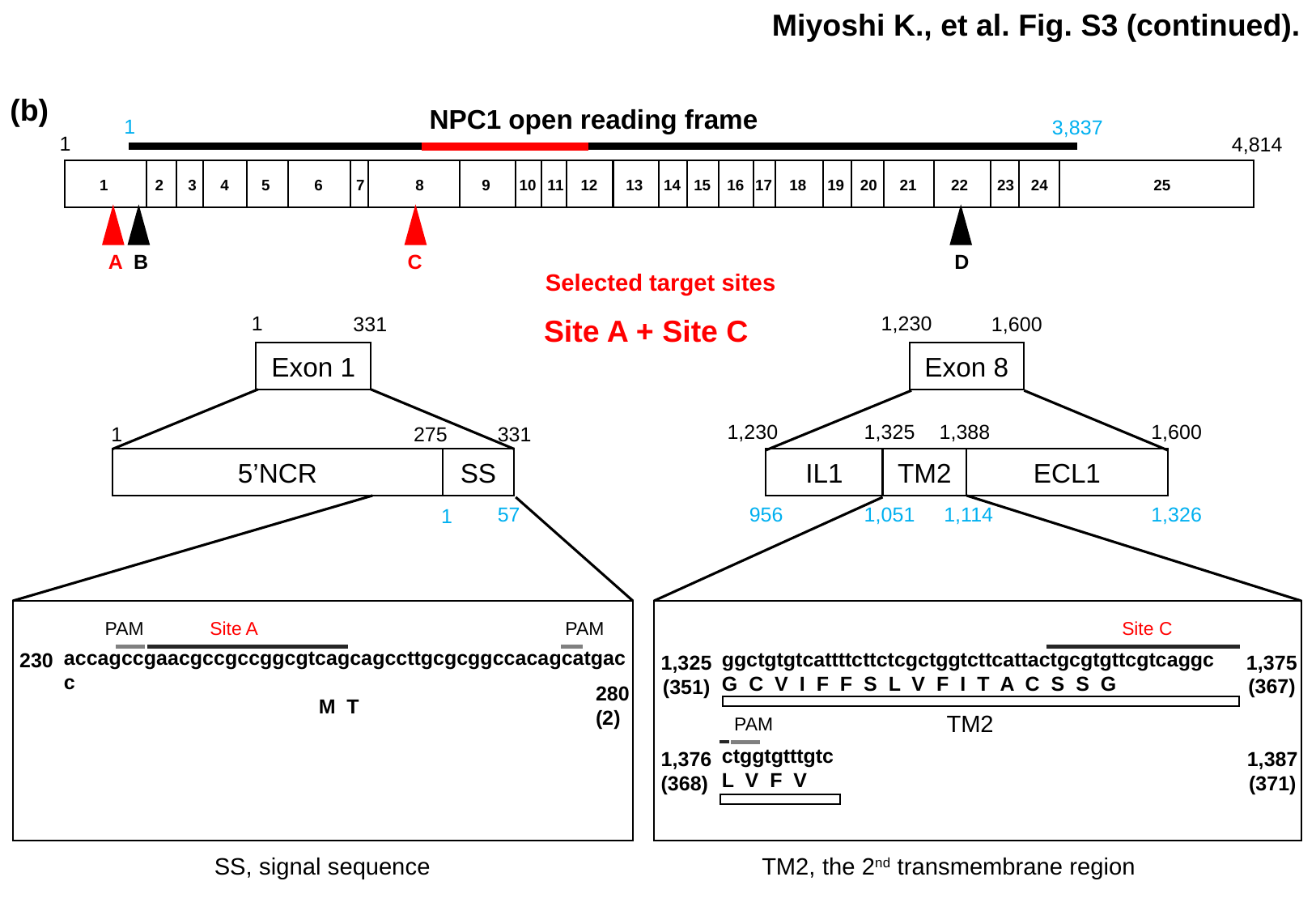

Miyoshi K., et al. Fig. S3 (continued).
(b)
NPC1 open reading frame
1
3,837
1
4,814
1
2
3
4
5
6
7
8
9
10
11
12
13
14
15
16
17
18
19
20
21
22
23
24
25
A
B
C
D
Selected target sites
1
1,230
331
1,600
Site A + Site C
Exon 1
Exon 8
1,230
1,325
1,388
1,600
1
275
331
SS
TM2
ECL1
5’NCR
IL1
57
956
1,051
1,114
1,326
1
Site A
PAM
Site C
PAM
accagccgaacgccgccggcgtcagcagccttgcgcggccacagcatgacc
 M T
ggctgtgtcattttcttctcgctggtcttcattactgcgtgttcgtcaggc
G C V I F F S L V F I T A C S S G
ctggtgtttgtc
L V F V
230
1,375
(367)
1,325
(351)
280
(2)
TM2
PAM
1,376
(368)
1,387
(371)
SS, signal sequence
TM2, the 2nd transmembrane region

## Slide 3
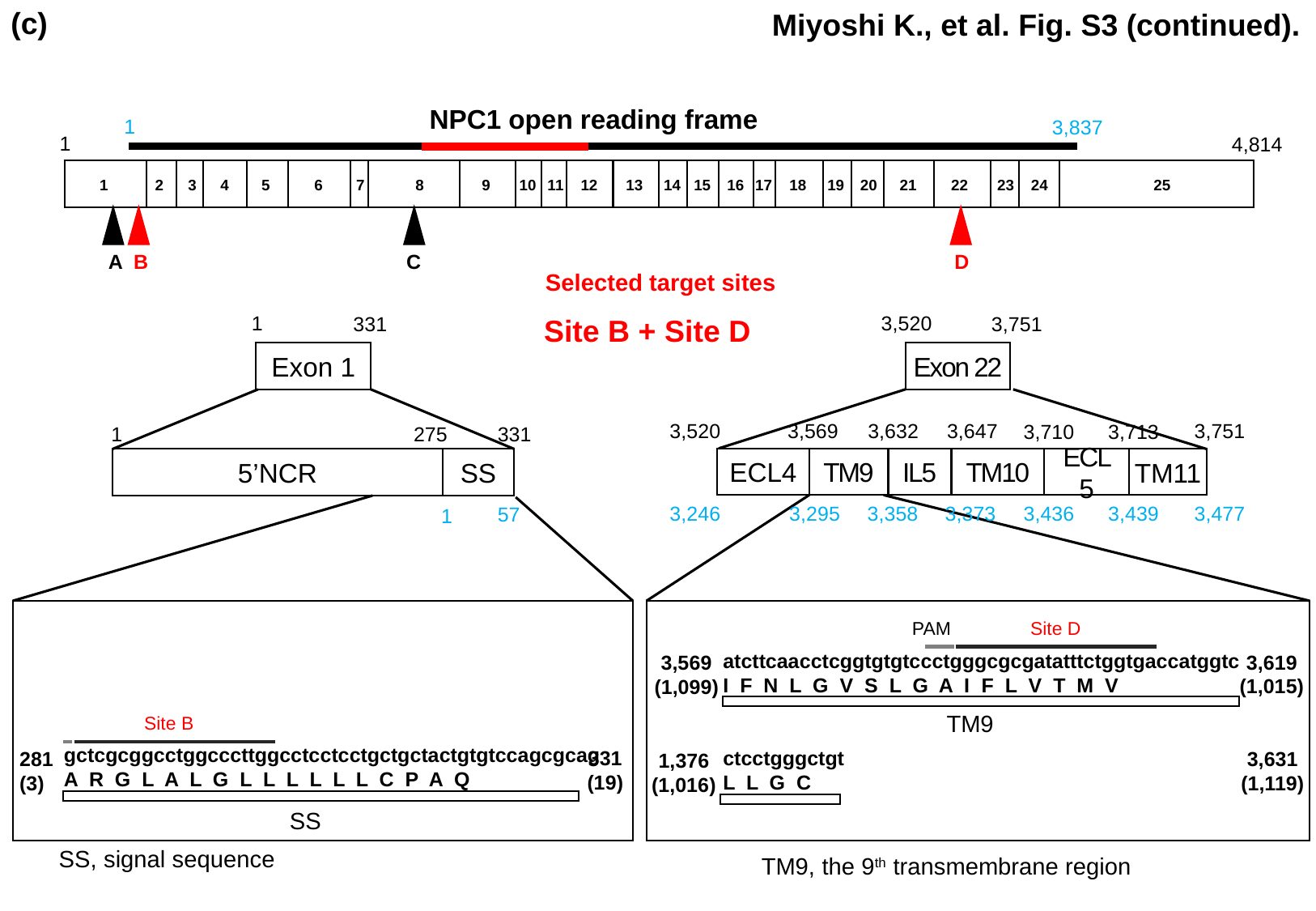

Miyoshi K., et al. Fig. S3 (continued).
(c)
NPC1 open reading frame
1
3,837
1
4,814
1
2
3
4
5
6
7
8
9
10
11
12
13
14
15
16
17
18
19
20
21
22
23
24
25
A
B
C
D
Selected target sites
1
3,520
331
3,751
Site B + Site D
Exon 1
Exon 22
3,520
3,569
3,632
3,647
3,751
3,710
3,713
1
275
331
ECL4
TM9
IL5
TM10
ECL5
TM11
SS
5’NCR
3,246
3,295
3,358
3,373
3,436
3,439
3,477
57
1
Site D
PAM
gctcgcggcctggcccttggcctcctcctgctgctactgtgtccagcgcag
A R G L A L G L L L L L L C P A Q
atcttcaacctcggtgtgtccctgggcgcgatatttctggtgaccatggtc
I F N L G V S L G A I F L V T M V
ctcctgggctgt
L L G C
3,619
(1,015)
3,569
(1,099)
TM9
Site B
331
(19)
281
(3)
3,631
(1,119)
1,376
(1,016)
SS
SS, signal sequence
TM9, the 9th transmembrane region
