# Supplementary material for: Global Proteomics for Identifying the Alteration Pathway of Niemann–Pick Disease Type C Using Hepatic Cell Models
Source: Int J Mol Sci. 2023 Oct 27;24(21):15642. doi: 10.3390/ijms242115642 (PMC10648601; doi:10.3390/ijms242115642)
Supplement: Supplementary file 1 [file ijms-24-15642-s001.zip › Figure S4_3.2.pptx]

## Slide 1
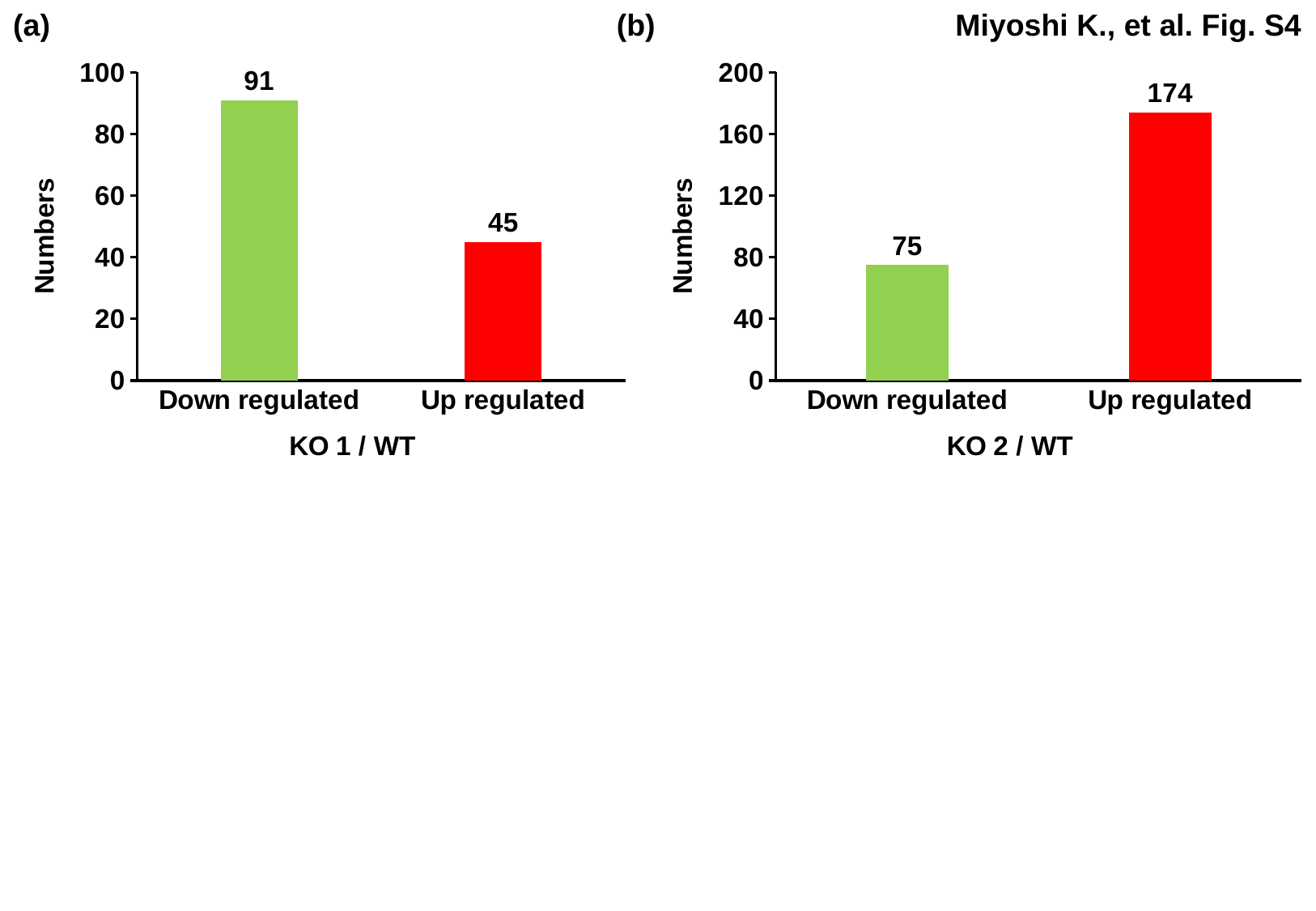

(a)
(b)
Miyoshi K., et al. Fig. S4
### Chart
| Category | |
|---|---|
| Down regulated | 75.0 |
| Up regulated | 174.0 |
### Chart
| Category | |
|---|---|
| Down regulated | 91.0 |
| Up regulated | 45.0 |
