# Supplementary material for: Global Proteomics for Identifying the Alteration Pathway of Niemann–Pick Disease Type C Using Hepatic Cell Models
Source: Int J Mol Sci. 2023 Oct 27;24(21):15642. doi: 10.3390/ijms242115642 (PMC10648601; doi:10.3390/ijms242115642)
Supplement: Supplementary file 1 [file ijms-24-15642-s001.zip › Figure S5_3.2.pptx]

## Slide 1
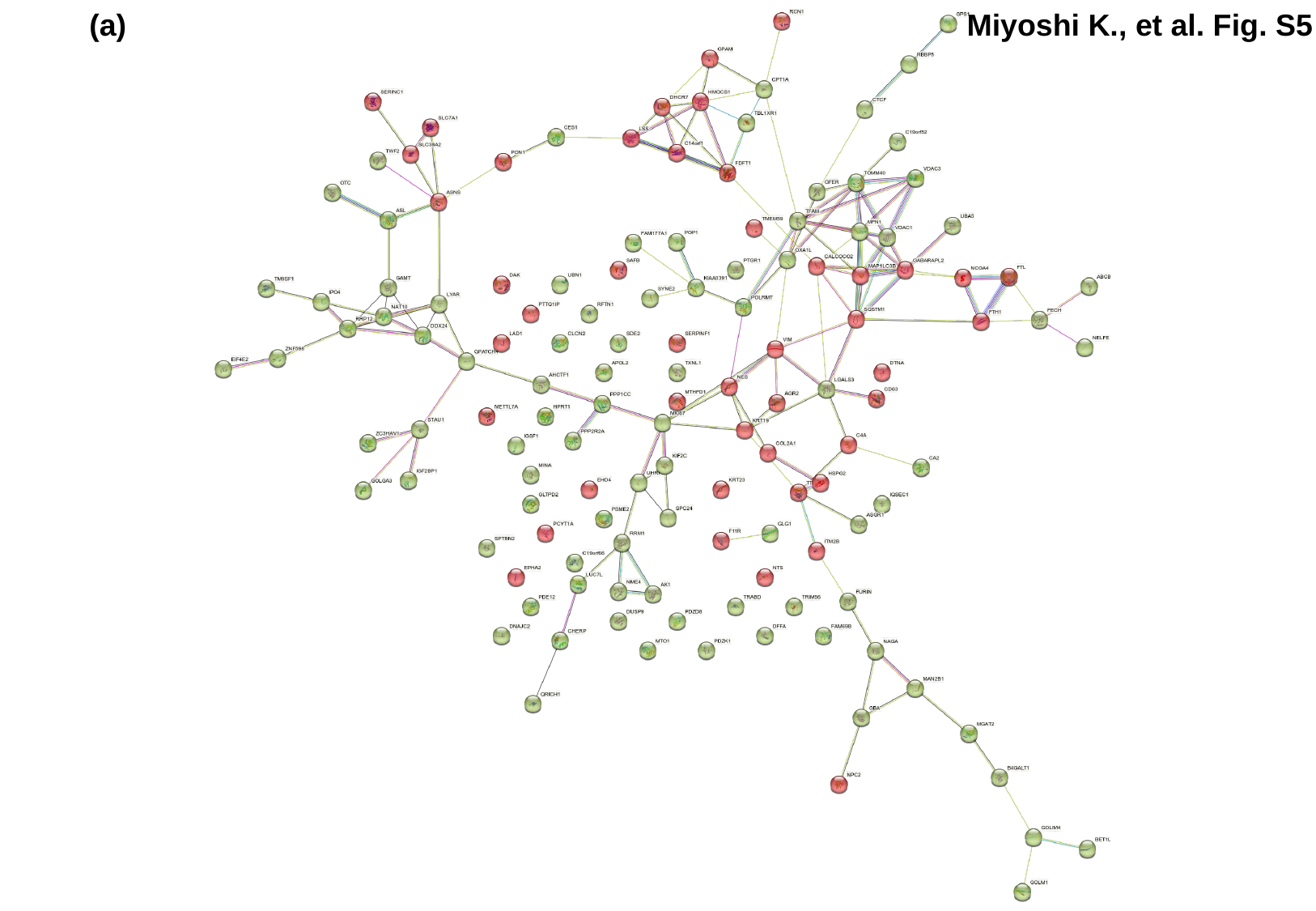

(a)
Miyoshi K., et al. Fig. S5

## Slide 2
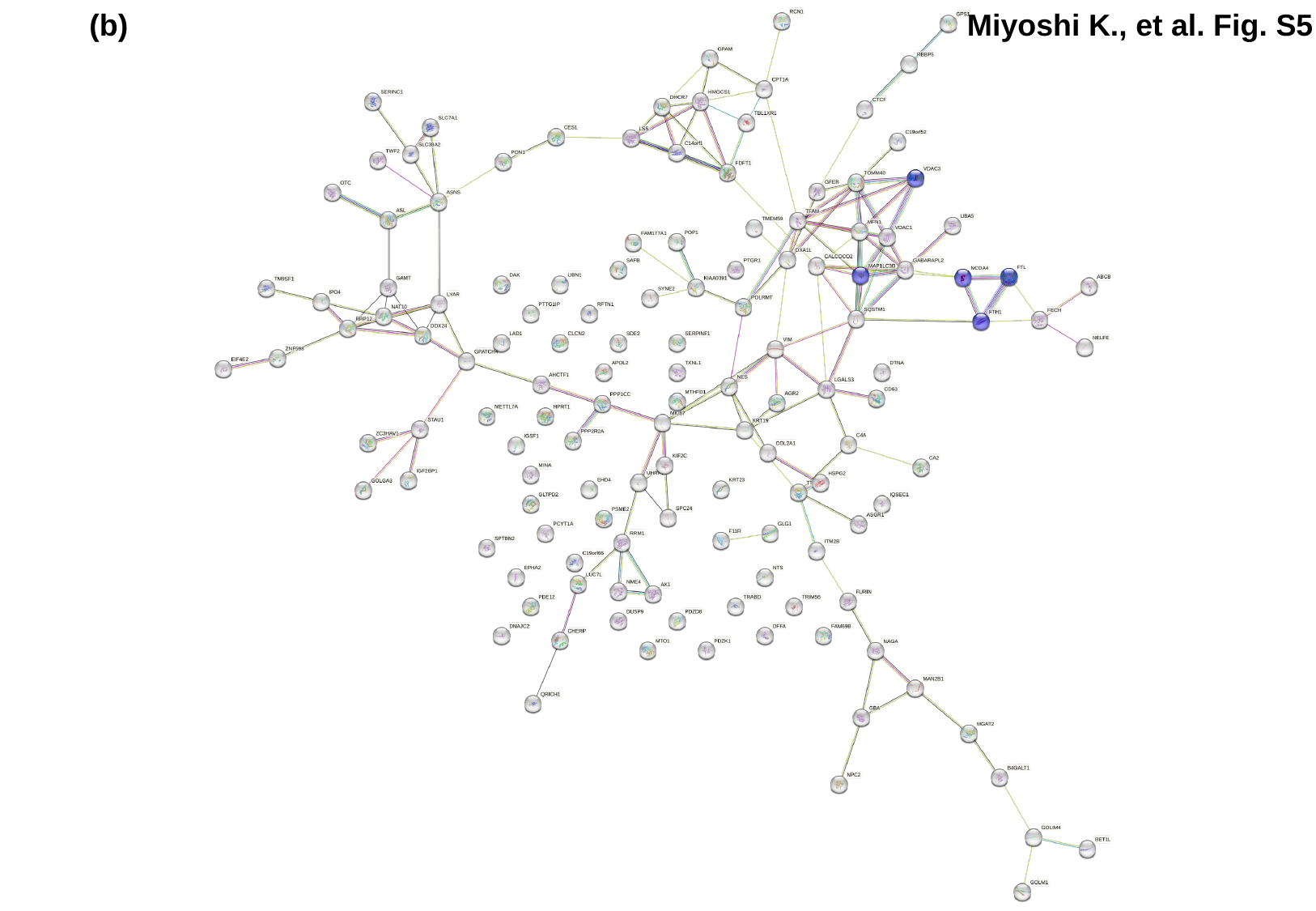

(b)
Miyoshi K., et al. Fig. S5

## Slide 3
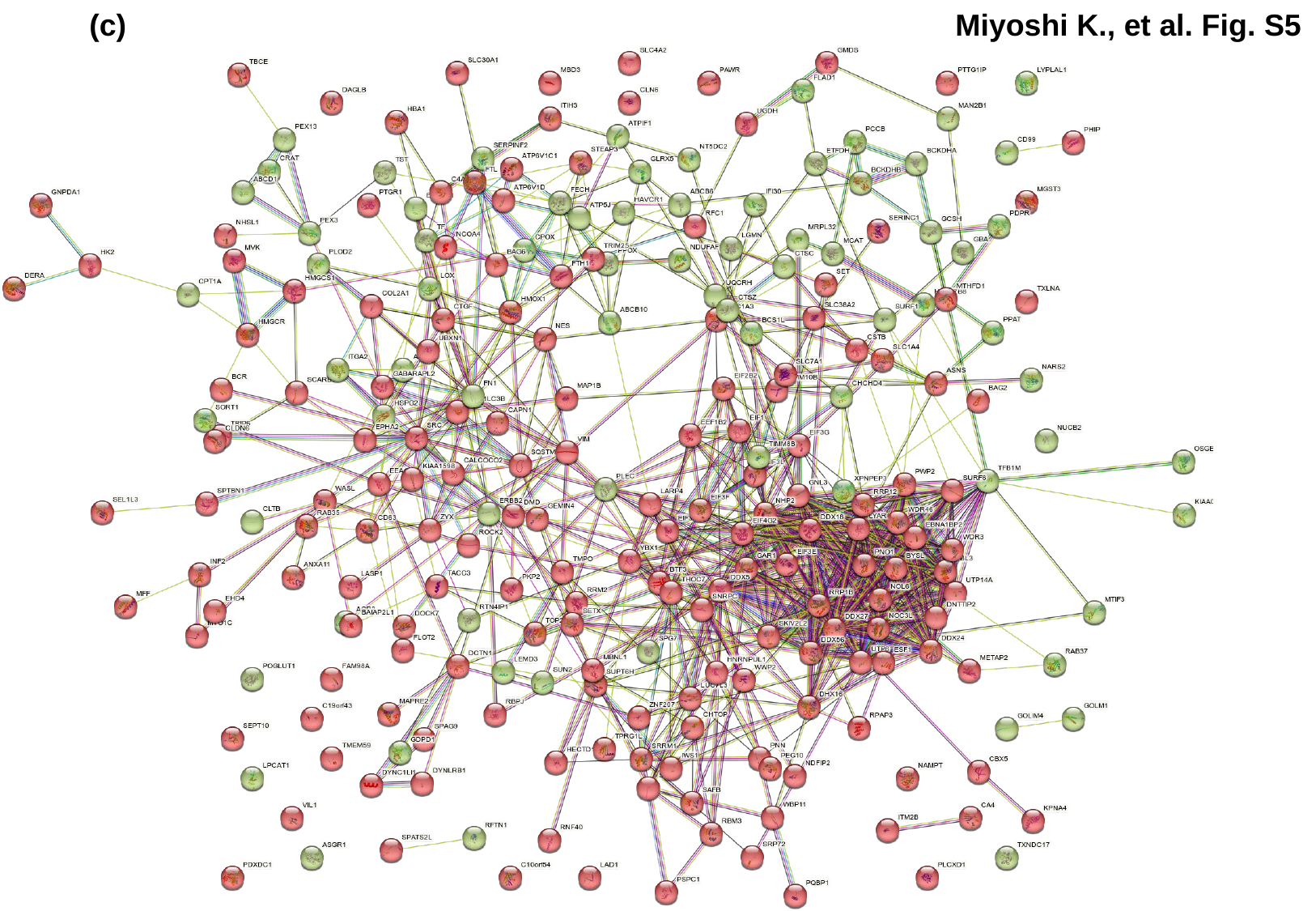

(c)
Miyoshi K., et al. Fig. S5

## Slide 4
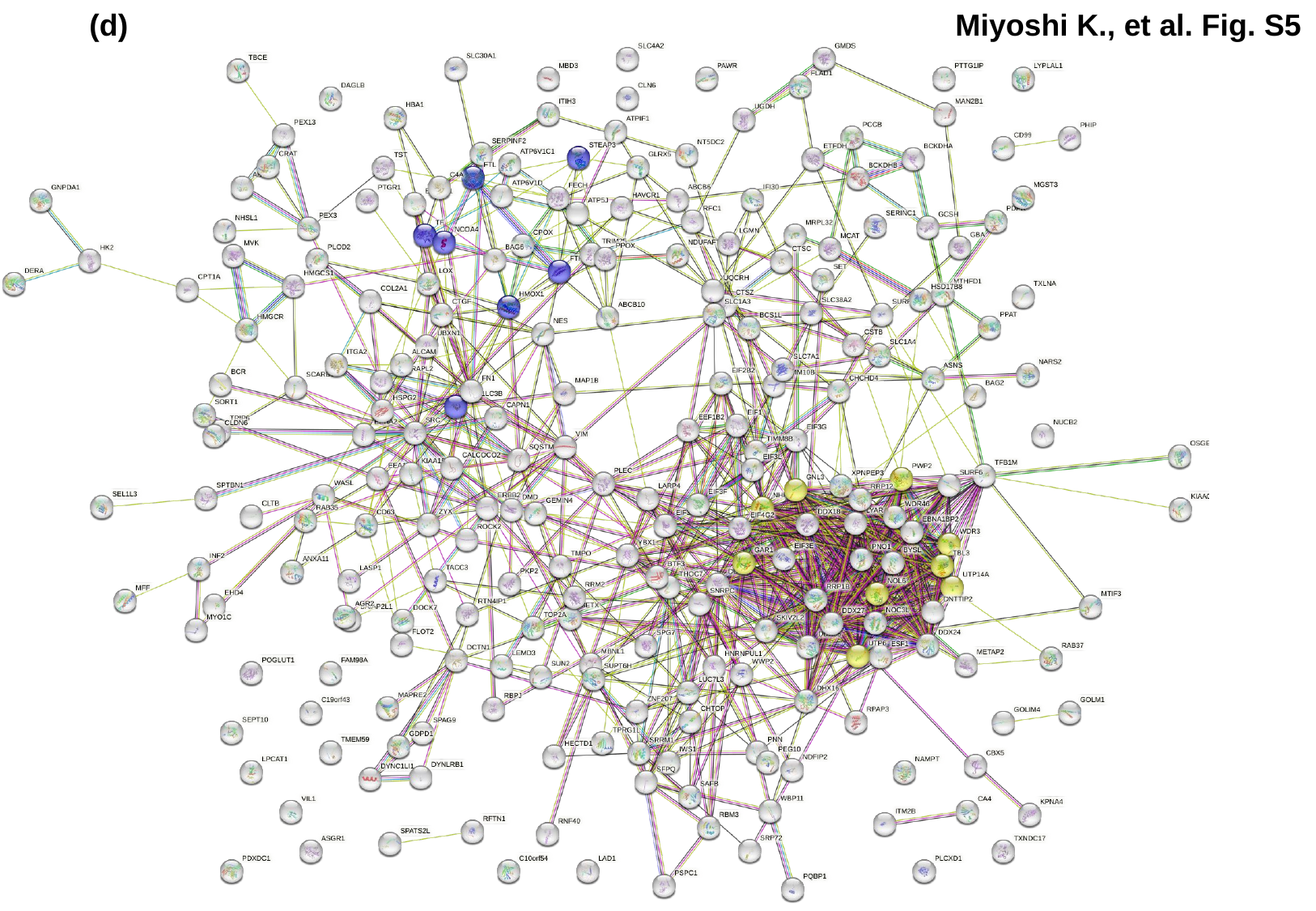

(d)
Miyoshi K., et al. Fig. S5
