# Supplementary material for: Global Proteomics for Identifying the Alteration Pathway of Niemann–Pick Disease Type C Using Hepatic Cell Models
Source: Int J Mol Sci. 2023 Oct 27;24(21):15642. doi: 10.3390/ijms242115642 (PMC10648601; doi:10.3390/ijms242115642)
Supplement: Supplementary file 1 [file ijms-24-15642-s001.zip › Figure S6_3.2.pptx]

## Slide 1
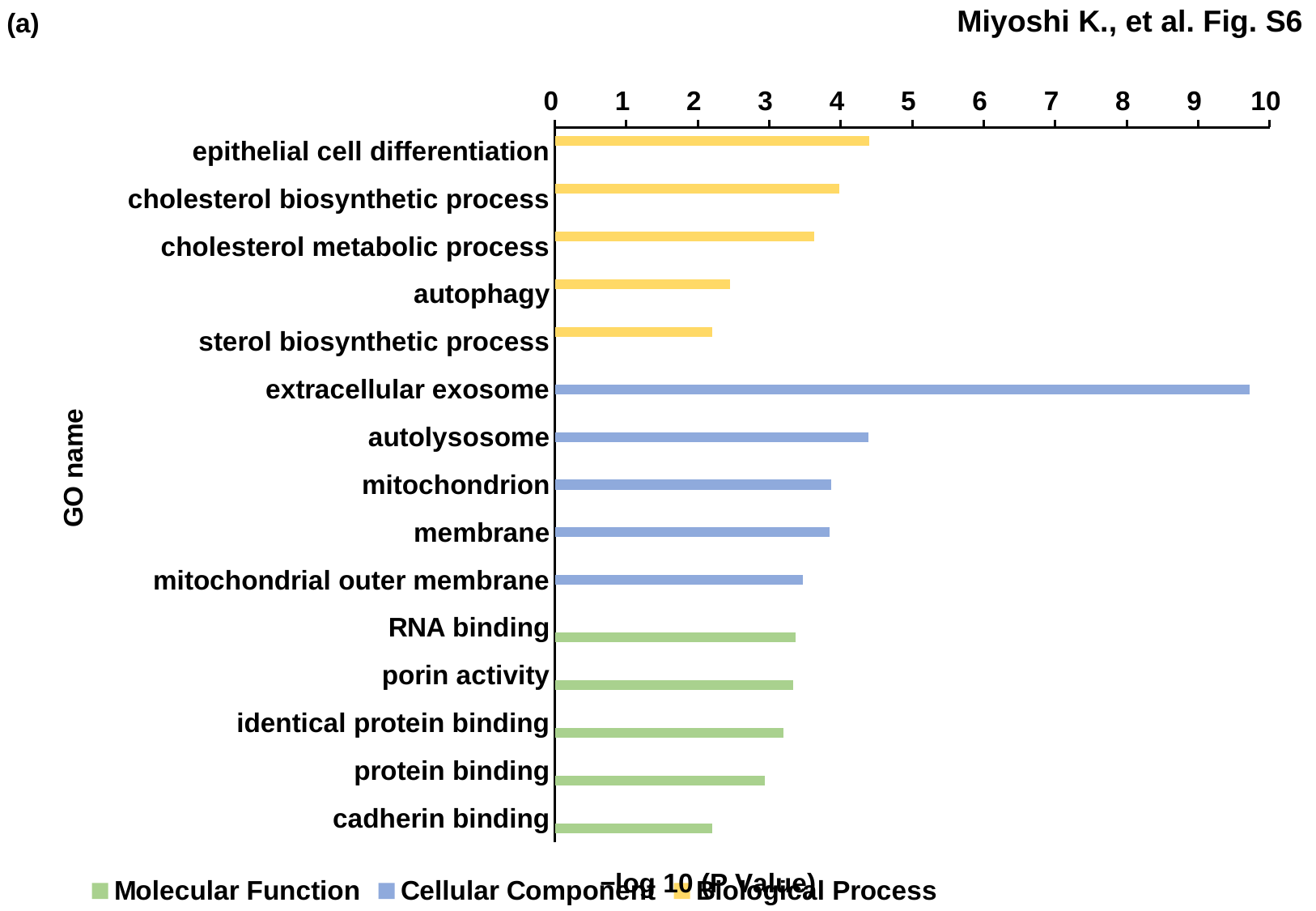

### Chart
| Category | | | |
|---|---|---|---|
| epithelial cell differentiation | 4.402794930911137 | None | None |
| cholesterol biosynthetic process | 3.9773763898512415 | None | None |
| cholesterol metabolic process | 3.630264869805133 | None | None |
| autophagy | 2.450454519791937 | None | None |
| sterol biosynthetic process | 2.200468199108381 | None | None |
| extracellular exosome | None | 9.722262553515904 | None |
| autolysosome | None | 4.387358801623152 | None |
| mitochondrion | None | 3.8709355432116213 | None |
| membrane | None | 3.8456051582985373 | None |
| mitochondrial outer membrane | None | 3.466902602321358 | None |
| RNA binding | None | None | 3.3709126943767544 |
| porin activity | None | None | 3.3346259916517345 |
| identical protein binding | None | None | 3.2015473969337642 |
| protein binding | None | None | 2.932742565842981 |
| cadherin binding | None | None | 2.2031521891022727 |(a)
Miyoshi K., et al. Fig. S6

## Slide 2
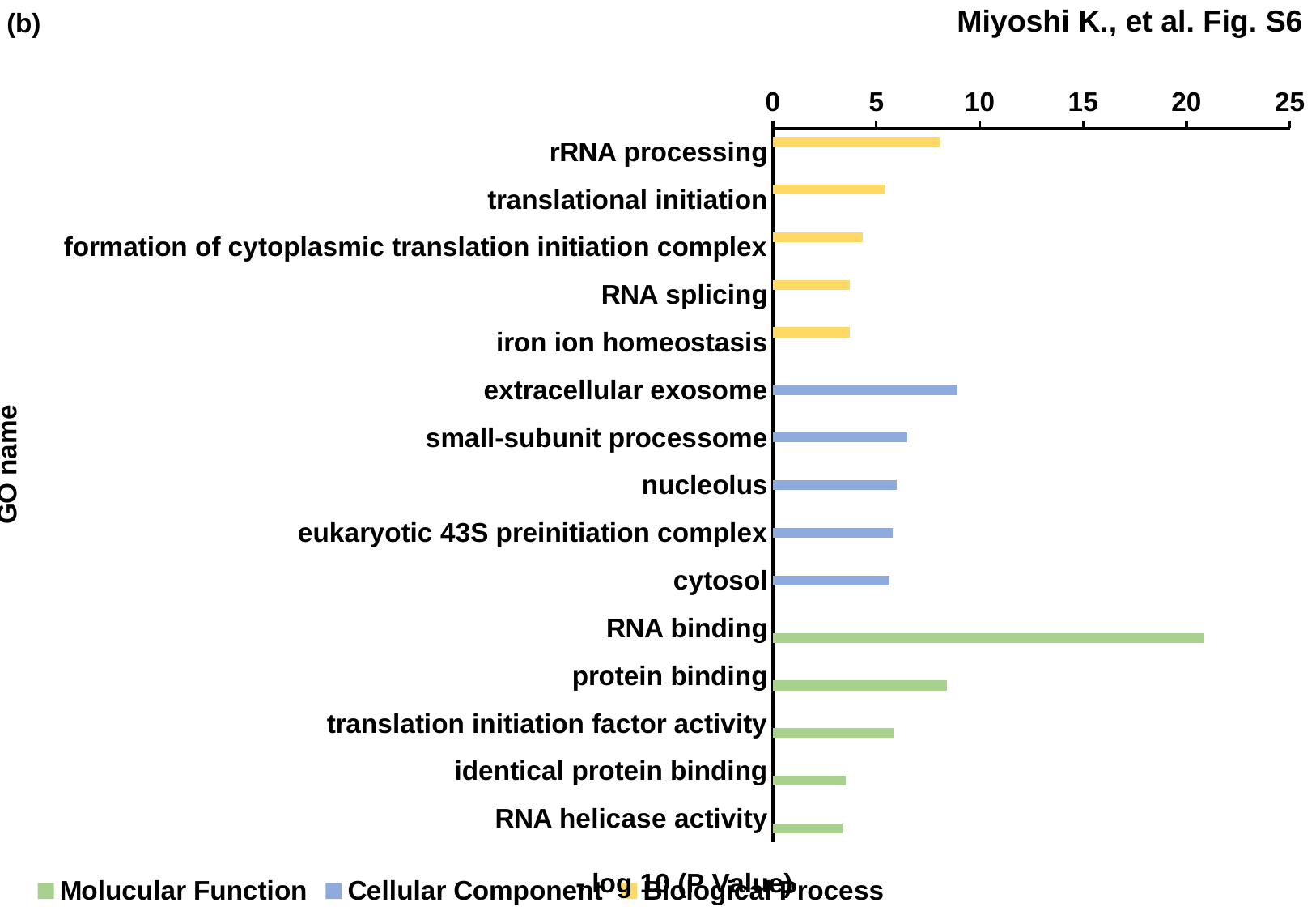

(b)
### Chart
| Category | | | |
|---|---|---|---|
| rRNA processing | 8.063351752799328 | None | None |
| translational initiation | 5.427770488524108 | None | None |
| formation of cytoplasmic translation initiation complex | 4.3280041535808405 | None | None |
| RNA splicing | 3.7106373364931504 | None | None |
| iron ion homeostasis | 3.701100885652968 | None | None |
| extracellular exosome | None | 8.910229099669836 | None |
| small-subunit processome | None | 6.4973533401592976 | None |
| nucleolus | None | 5.968508342591756 | None |
| eukaryotic 43S preinitiation complex | None | 5.787496109261197 | None |
| cytosol | None | 5.61843470342385 | None |
| RNA binding | None | None | 20.85751050818424 |
| protein binding | None | None | 8.412980060295242 |
| translation initiation factor activity | None | None | 5.828616256570158 |
| identical protein binding | None | None | 3.530672182051249 |
| RNA helicase activity | None | None | 3.34789056298269 |Miyoshi K., et al. Fig. S6
