# Supplementary material for: Global Proteomics for Identifying the Alteration Pathway of Niemann–Pick Disease Type C Using Hepatic Cell Models
Source: Int J Mol Sci. 2023 Oct 27;24(21):15642. doi: 10.3390/ijms242115642 (PMC10648601; doi:10.3390/ijms242115642)
Supplement: Supplementary file 1 [file ijms-24-15642-s001.zip › Table S1_3.2.pdf]

Table S1 Identified protein numbers in global label free proteomics.

Region	Group	Exclusive (number)	Total (number)
A	WT	37	3530
B	KO1	24	3468
C	KO2	82	3570
D	WT and KO1	59	3608
E	KO1 and KO2	54	3653
F	WT and KO2	103	3666
G	WT and KO1 and KO2	3331	3331
H	Sum	3690	

WT, hep G2 cells; KO1, site A and C mutant NPC1 model cells; KO1, site B and D mutant NPC1 model cells.
