# Supplementary material for: Global Proteomics for Identifying the Alteration Pathway of Niemann–Pick Disease Type C Using Hepatic Cell Models
Source: Int J Mol Sci. 2023 Oct 27;24(21):15642. doi: 10.3390/ijms242115642 (PMC10648601; doi:10.3390/ijms242115642)
Supplement: Supplementary file 1 [file ijms-24-15642-s001.zip › Table S3_3.2.pdf]

Table S3 Differentially expression proteins in label free global proteomics

(a) Up-regulated proteins comparison KO1 versus WT

**Accession**	**Description**	**Gene Symbol**	**Fold change**	***P*-value**
P02458	Collagen alpha-1(II) chain	COL2A1	24.03	4.00E-06
Q13772	Nuclear receptor coactivator 4	NCOA4	8.75	3.15E-05
P08670	Vimentin	VIM	4.94	2.46E-11
Q3LXA3	Triokinase/FMN cyclase	TKFC	4.90	1.40E-04
O95994	Anterior gradient protein 2 homolog	AGR2	4.86	2.00E-08
Q13137	Calcium-binding and coiled-coil domain-containing protein 2	CALCOCO2	4.57	3.68E-06
P60520	Gamma-aminobutyric acid receptor-associated protein-like 2	GABARAPL2	4.32	2.14E-04
Q9H223	EH domain-containing protein 4	EHD4	4.27	8.61E-06
P08727	Keratin, type I cytoskeletal 19	KRT19	4.19	3.08E-10
P48681	Nestin	NES	4.07	1.97E-10
O00515	Ladinin-1	LAD1	3.41	5.92E-03
Q15293	Reticulocalbin-1	RCN1	3.36	3.17E-05
Q96QD8	Sodium-coupled neutral amino acid transporter 2	SLC38A2	3.17	1.35E-05
P02766	Transthyretin	TTR	3.13	1.68E-05
P11586	C-1-tetrahydrofolate synthase, cytoplasmic	MTHFD1	2.97	1.63E-06
P02794	Ferritin heavy chain	FTH1	2.96	8.39E-04
P30990	Neurotensin/neuromedin N	NTS	2.87	5.80E-08
P08962	CD63 antigen	CD63	2.84	6.54E-09
P27169	Serum paraoxonase/arylesterase 1	PON1	2.77	1.44E-04
P0C0L4	Complement C4-A	C4A	2.71	1.11E-03

Accession	Description	Gene Symbol	Fold change	*P*-value
Q15424	Scaffold attachment factor B1	SAFB	2.68	1.43E-06
Q9HCL2	Glycerol-3-phosphate acyltransferase 1, mitochondrial	GPAM	2.59	7.90E-03
Q9Y287	Integral membrane protein 2B	ITM2B	2.58	2.83E-03
P08243	Asparagine synthetase [glutamine-hydrolyzing]	ASNS	2.56	1.66E-04
P37268	Squalene synthase	FDFT1	2.55	5.58E-10
P30825	High affinity cationic amino acid transporter 1	SLC7A1	2.46	8.18E-09
Q9GZQ8	Microtubule-associated proteins 1A/1B light chain 3B	MAP1LC3B	2.39	1.40E-06
P02792	Ferritin light chain	FTL	2.37	3.72E-05
Q9Y4J8	Dystrobrevin alpha	DTNA	2.36	5.28E-05
P53801	Pituitary tumor-transforming gene 1 protein-interacting protein	PTTG1IP	2.34	4.24E-05
P48449	Lanosterol synthase	LSS	2.33	1.78E-09
Q9UKR5	Ergosterol biosynthetic protein 28 homolog	ERG28	2.30	1.24E-02
Q9UBM7	7-dehydrocholesterol reductase	DHCR7	2.28	3.84E-07
Q9C075	Keratin, type I cytoskeletal 23	KRT23	2.26	1.75E-06
Q9NRX5	Serine incorporator 1	SERINC1	2.17	7.86E-05
Q9H8H3	Putative methyltransferase-like protein 7A	METTL7A	2.14	1.08E-04
Q9Y624	Junctional adhesion molecule A	F11R	2.13	8.61E-07
P36955	Pigment epithelium-derived factor	SERPINF1	2.11	4.40E-04
P61916	NPC intracellular cholesterol transporter 2	NPC2	2.09	1.80E-08
P49585	Choline-phosphate cytidylyltransferase A	PCYT1A	2.07	4.46E-05
Q9BXS4	Transmembrane protein 59	TMEM59	2.06	4.19E-05
Q13501	Sequestosome-1	SQSTM1	2.05	1.76E-05

Accession	Description	Gene Symbol	Fold change	*P*-value
Q01581	Hydroxymethylglutaryl-CoA synthase, cytoplasmic	HMGCS1	2.05	3.99E-06
P98160	Basement membrane-specific heparan sulfate proteoglycan core protein	HSPG2	2.03	1.05E-05
P29317	Ephrin type-A receptor 2	EPHA2	2.02	4.03E-07

(b) Down-regulated proteins comparison KO1 versus WT

Accession	Description	Gene Symbol	Fold change	*P*-value
O00273	DNA fragmentation factor subunit alpha	DFFA	0.050	3.24E-02
Q96T88	E3 ubiquitin-protein ligase UHRF1	UHRF1	0.102	2.71E-03
Q14699	Raftlin	RFTN1	0.145	2.50E-05
P23141	Liver carboxylesterase 1	CES1	0.158	7.00E-08
O00746	Nucleoside diphosphate kinase, mitochondrial	NME4	0.209	1.24E-04
Q14914	Prostaglandin reductase 1	PTGR1	0.210	9.37E-05
P50416	Carnitine O-palmitoyltransferase 1, liver isoform	CPT1A	0.226	9.75E-09
Q8TEX9	Importin-4	IPO4	0.226	4.69E-04
Q9GZZ9	Ubiquitin-like modifier-activating enzyme 5	UBA5	0.229	4.84E-03
P04062	Lysosomal acid glucosylceramidase	GBA	0.246	5.68E-08
Q9NUL5	Shiftless antiviral inhibitor of ribosomal frameshifting protein	SHFL	0.250	3.64E-04
Q99956	Dual specificity protein phosphatase 9	DUSP9	0.281	4.84E-04
O43396	Thioredoxin-like protein 1	TXNL1	0.298	3.02E-03
Q9UL46	Proteasome activator complex subunit 2	PSME2	0.313	1.88E-02
Q9BSF4	Mitochondrial import inner membrane translocase subunit Tim29	TIMM29	0.316	7.45E-03
Q5T2W1	Na(+)/H(+) exchange regulatory cofactor NHE-RF3	PDZK1	0.321	6.97E-03

Accession	Description	Gene Symbol	Fold change	*P*-value
P51788	Chloride channel protein 2	CLCN2	0.324	1.37E-03
Q9Y2Z2	Protein MTO1 homolog, mitochondrial	MTO1	0.326	8.63E-03
Q00059	Transcription factor A, mitochondrial	TFAM	0.326	1.12E-02
Q9BZK7	F-box-like/WD repeat-containing protein TBL1XR1	TBL1XR1	0.330	4.76E-04
P09958	Furin	FURIN	0.332	3.88E-03
O15091	Mitochondrial ribonuclease P catalytic subunit	PRORP	0.334	9.28E-05
Q13098	COP9 signalosome complex subunit 1	GPS1	0.334	6.56E-04
P49711	Transcriptional repressor CTCF	CTCF	0.335	4.02E-02
Q8IWX8	Calcium homeostasis endoplasmic reticulum protein	CHERP	0.348	2.37E-03
Q8IWA4	Mitofusin-1	MFN1	0.358	3.48E-04
Q8NBJ4	Golgi membrane protein 1	GOLM1	0.361	3.76E-07
Q8WXH0	Nesprin-2	SYNE2	0.363	4.87E-04
P18615	Negative elongation factor E	NELFE	0.370	1.88E-03
Q8IUF8	Ribosomal oxygenase 2	RIOX2	0.372	4.76E-03
P22830	Ferrochelatase, mitochondrial	FECH	0.374	2.50E-06
P00480	Ornithine transcarbamylase, mitochondrial	OTC	0.376	1.17E-05
O95793	Double-stranded RNA-binding protein Staufen homolog 1	STAU1	0.377	7.21E-03
Q7Z2W4	Zinc finger CCCH-type antiviral protein 1	ZC3HAV1	0.390	1.42E-03
Q5T3I0	G patch domain-containing protein 4	GPATCH4	0.394	9.24E-03
O00754	Lysosomal alpha-mannosidase	MAN2B1	0.396	4.24E-08
O00461	Golgi integral membrane protein 4	GOLIM4	0.396	2.70E-05
Q9NX58	Cell growth-regulating nucleolar protein	LYAR	0.398	4.58E-02

**Accession**	**Description**	**Gene Symbol**	**Fold change**	***P*-value**
Q8NEN9	PDZ domain-containing protein 8	PDZD8	0.399	5.08E-03
Q9NP58	ATP-binding cassette sub-family B member 6	ABCB6	0.400	1.16E-03
Q6DN90	IQ motif and SEC7 domain-containing protein 1	IQSEC1	0.400	1.97E-04
Q9H4I3	TraB domain-containing protein	TRABD	0.402	5.42E-05
Q8N128	Protein FAM177A1	FAM177A1	0.403	1.53E-03
Q6L8Q7	2',5'-phosphodiesterase 12	PDE12	0.412	2.27E-02
Q8WYP5	Protein ELYS	AHCTF1	0.415	3.42E-05
Q9GZR7	ATP-dependent RNA helicase DDX24	DDX24	0.416	1.68E-02
Q9Y277	Voltage-dependent anion-selective channel protein 3	VDAC3	0.417	4.39E-03
P46013-2	Isoform Short of Proliferation marker protein Ki-67	MKI67	0.419	1.50E-05
Q8NBT2	Kinetochore protein Spc24	SPC24	0.422	3.13E-05
P21796	Voltage-dependent anion-selective channel protein 1	VDAC1	0.426	1.80E-04
Q15070	Mitochondrial inner membrane protein OXA1L	OXA1L	0.426	1.61E-02
O96008	Mitochondrial import receptor subunit TOM40 homolog	TOMM40	0.431	1.63E-02
P17050	Alpha-N-acetylgalactosaminidase	NAGA	0.433	5.36E-05
Q9NZI8	Insulin-like growth factor 2 mRNA-binding protein 1	IGF2BP1	0.433	4.00E-02
O15321	Transmembrane 9 superfamily member 1	TM9SF1	0.435	3.23E-04
P17931	Galectin-3	LGALS3	0.438	1.66E-04
Q14353	Guanidinoacetate N-methyltransferase	GAMT	0.439	4.17E-02
Q15291	Retinoblastoma-binding protein 5	RBBP5	0.446	6.01E-03
P07306	Asialoglycoprotein receptor 1	ASGR1	0.446	1.89E-05
P15291	Beta-1,4-galactosyltransferase 1	B4GALT1	0.449	8.33E-05

Accession	Description	Gene Symbol	Fold change	*P*-value
A6NH11	Glycolipid transfer protein domain-containing protein 2	GLTPD2	0.449	1.68E-05
Q6IQ49	Replication stress response regulator SDE2	SDE2	0.449	3.74E-02
P04424	Argininosuccinate lyase	ASL	0.449	1.04E-02
P36873	Serine/threonine-protein phosphatase PP1-gamma catalytic subunit	PPP1CC	0.450	7.26E-04
Q9BQE5	Apolipoprotein L2	APOL2	0.450	5.30E-05
Q10469	Alpha-1,6-mannosyl-glycoprotein 2-beta-N-acetylglucosaminyltransferase	MGAT2	0.450	2.84E-05
Q2TAL8	Transcriptional regulator QRICH1	QRICH1	0.453	5.57E-03
Q08378	Golgin subfamily A member 3	GOLGA3	0.456	2.07E-03
Q9NQ29	Putative RNA-binding protein Luc7-like 1	LUC7L	0.457	2.10E-03
Q9BRZ2	E3 ubiquitin-protein ligase TRIM56	TRIM56	0.459	3.02E-03
Q99661	Kinesin-like protein KIF2C	KIF2C	0.460	1.68E-03
O60573	Eukaryotic translation initiation factor 4E type 2	EIF4E2	0.460	2.45E-02
P00568	Adenylate kinase isoenzyme 1	AK1	0.460	2.52E-02
P00918	Carbonic anhydrase 2	CA2	0.461	9.01E-03
P55789	FAD-linked sulfhydryl oxidase ALR	GFER	0.462	1.13E-03
Q92896	Golgi apparatus protein 1	GLG1	0.466	1.17E-06
Q5JTH9	RRP12-like protein	RRP12	0.471	3.76E-02
Q99575	Ribonucleases P/MRP protein subunit POP1	POP1	0.472	3.03E-02
P63151	Serine/threonine-protein phosphatase 2A 55 kDa regulatory subunit B alpha isoform	PPP2R2A	0.473	1.51E-03
Q9NYM9	BET1-like protein	BET1L	0.475	3.97E-03
Q5VUD6	Divergent protein kinase domain 1B	DIPK1B	0.479	1.53E-02
O15020	Spectrin beta chain, non-erythrocytic 2	SPTBN2	0.481	3.90E-03

Accession	Description	Gene Symbol	Fold change	*P*-value
O00411	DNA-directed RNA polymerase, mitochondrial	POLRMT	0.490	4.09E-04
Q9H0A0	RNA cytidine acetyltransferase	NAT10	0.492	2.31E-03
P00492	Hypoxanthine-guanine phosphoribosyltransferase	HPRT1	0.495	7.05E-03
Q86UK7	E3 ubiquitin-protein ligase ZNF598	ZNF598	0.496	4.60E-03
Q9NPG3	Ubinuclein-1	UBN1	0.496	4.17E-04
Q99543	DnaJ homolog subfamily C member 2	DNAJC2	0.497	3.92E-02
Q8N6C5	Immunoglobulin superfamily member 1	IGSF1	0.497	1.35E-04
P23921	Ribonucleoside-diphosphate reductase large subunit	RRM1	0.498	1.63E-02
Q6IBS0	Twinfilin-2	TWF2	0.498	1.69E-02

(c) Up-regulated proteins comparison KO2 versus WT

Accession	Description	Gene Symbol	Fold change	*P*-value
P02458	Collagen alpha-1(II) chain	COL2A1	37.8	8.14E-07
Q13772	Nuclear receptor coactivator 4	NCOA4	16.2	2.91E-08
Q13137	Calcium-binding and coiled-coil domain-containing protein 2	CALCOCO2	7.99	1.82E-08
P09234	U1 small nuclear ribonucleoprotein C	SNRPC	5.88	1.73E-02
Q13501	Sequestosome-1	SQSTM1	5.83	1.50E-08
P02794	Ferritin heavy chain	FTH1	5.82	3.71E-07
Q9H223	EH domain-containing protein 4	EHD4	5.48	3.99E-05
P43003	Excitatory amino acid transporter 1	SLC1A3	5.31	2.58E-06
P02792	Ferritin light chain	FTL	5.19	7.22E-09
P60520	Gamma-aminobutyric acid receptor-associated protein-like 2	GABARAPL2	4.96	7.04E-03

Accession	Description	Gene Symbol	Fold change	*P*-value
O00515	Ladinin-1	LAD1	4.69	2.65E-04
Q06033	Inter-alpha-trypsin inhibitor heavy chain H3	ITIH3	4.59	5.14E-07
Q06330	Recombining binding protein suppressor of hairless	RBPJ	4.01	2.87E-07
Q15942	Zyxin	ZYX	3.88	4.82E-04
P0C0L4	Complement C4-A	C4A	3.87	6.43E-04
Q7KZ85	Transcription elongation factor SPT6	SUPT6H	3.77	2.03E-05
Q9BVJ6	U3 small nucleolar RNA-associated protein 14 homolog A	UTP14A	3.73	2.67E-04
P09327	Villin-1	VIL1	3.67	3.28E-07
P04920	Anion exchange protein 2	SLC4A2	3.52	3.38E-07
Q9NUJ7	PI-PLC X domain-containing protein 1	PLCXD1	3.50	1.39E-04
Q658P3	Metalloreductase STEAP3	STEAP3	3.47	5.74E-05
Q9BXS4	Transmembrane protein 59	TMEM59	3.46	4.14E-08
Q9GZQ8	Microtubule-associated proteins 1A/1B light chain 3B	MAP1LC3B	3.44	1.26E-07
Q9GZR7	ATP-dependent RNA helicase DDX24	DDX24	3.41	1.00E-05
Q9NV92	NEDD4 family-interacting protein 2	NDFIP2	3.38	2.92E-06
Q14258	E3 ubiquitin/ISG15 ligase TRIM25	TRIM25	3.38	3.04E-04
Q01105	Protein SET	SET	3.36	1.91E-06
P46926	Glucosamine-6-phosphate isomerase 1	GNPDA1	3.35	3.29E-06
Q5QJE6	Deoxynucleotidyltransferase terminal-interacting protein 2	DNTTIP2	3.31	3.96E-05
O15213	WD repeat-containing protein 46	WDR46	3.30	3.40E-05
Q01082-3	Isoform 2 of Spectrin beta chain, non-erythrocytic 1	SPTBN1	3.24	3.41E-06
O00308	NEDD4-like E3 ubiquitin-protein ligase WWP2	WWP2	3.20	7.31E-03

Accession	Description	Gene Symbol	Fold change	*P*-value
Q9UHR4	Brain-specific angiogenesis inhibitor 1-associated protein 2-like protein 1	BAIAP2L1	3.17	1.01E-03
Q68CR1	Protein sel-1 homolog 3	SEL1L3	3.17	1.21E-04
P08962	CD63 antigen	CD63	3.14	4.80E-09
P98179	RNA-binding protein 3	RBM3	3.12	3.91E-03
P29317	Ephrin type-A receptor 2	EPHA2	3.10	1.10E-08
P08243	Asparagine synthetase [glutamine-hydrolyzing]	ASNS	3.06	4.60E-04
Q6I9Y2	THO complex subunit 7 homolog	THOC7	3.03	1.56E-03
P22748	Carbonic anhydrase 4	CA4	3.03	3.99E-07
Q15813	Tubulin-specific chaperone E	TBCE	3.03	4.64E-02
P31350	Ribonucleoside-diphosphate reductase subunit M2	RRM2	2.99	1.35E-04
P04035	3-hydroxy-3-methylglutaryl-coenzyme A reductase	HMGCR	2.90	1.81E-07
P11532	Dystrophin	DMD	2.89	1.68E-07
Q13895	Bystin	BYSL	2.82	4.61E-03
Q9Y287	Integral membrane protein 2B	ITM2B	2.82	2.83E-03
O75821	Eukaryotic translation initiation factor 3 subunit G	EIF3G	2.82	9.83E-08
Q8NCA5	Protein FAM98A	FAM98A	2.81	1.11E-02
Q01581	Hydroxymethylglutaryl-CoA synthase, cytoplasmic	HMGCS1	2.81	9.56E-07
Q8WTT2	Nucleolar complex protein 3 homolog	NOC3L	2.79	3.84E-04
O60231	Pre-mRNA-splicing factor ATP-dependent RNA helicase DHX16	DHX16	2.75	4.46E-03
Q14914	Prostaglandin reductase 1	PTGR1	2.73	7.35E-05
P11274	Breakpoint cluster region protein	BCR	2.72	8.58E-03
Q96IZ0	PRKC apoptosis WT1 regulator protein	PAWR	2.70	4.37E-04

Accession	Description	Gene Symbol	Fold change	*P*-value
P56747	Claudin-6	CLDN6	2.67	7.42E-05
P53801	Pituitary tumor-transforming gene 1 protein-interacting protein	PTTG1IP	2.66	1.13E-07
Q99959	Plakophilin-2	PKP2	2.65	1.02E-04
P49770	Translation initiation factor eIF-2B subunit beta	EIF2B2	2.64	2.29E-03
Q9H501	ESF1 homolog	ESF1	2.55	5.71E-03
Q9NRX5	Serine incorporator 1	SERINC1	2.53	6.01E-06
Q9H6T3	RNA polymerase II-associated protein 3	RPAP3	2.51	3.25E-03
P67809	Y-box-binding protein 1	YBX1	2.50	1.63E-05
P08670	Vimentin	VIM	2.50	2.16E-09
Q9NX24	H/ACA ribonucleoprotein complex subunit 2	NHP2	2.49	1.43E-02
P50995	Annexin A11	ANXA11	2.49	1.39E-06
Q96GQ7	Probable ATP-dependent RNA helicase DDX27	DDX27	2.44	5.84E-05
Q8IYB3	Serine/arginine repetitive matrix protein 1	SRRM1	2.44	2.03E-04
P04080	Cystatin-B	CSTB	2.44	7.97E-03
O43670	BUB3-interacting and GLEBS motif-containing protein ZNF207	ZNF207	2.43	2.19E-04
Q9H307	Pinin	PNN	2.43	6.12E-03
O95816	BAG family molecular chaperone regulator 2	BAG2	2.43	3.37E-06
Q9NYH9	U3 small nucleolar RNA-associated protein 6 homolog	UTP6	2.42	1.84E-03
Q9ULT8	E3 ubiquitin-protein ligase HECTD1	HECTD1	2.42	1.03E-04
Q9NWW5	Ceroid-lipofuscinosis neuronal protein 6	CLN6	2.41	1.89E-04
P48681	Nestin	NES	2.39	7.37E-09
Q15269	Periodic tryptophan protein 2 homolog	PWP2	2.38	2.24E-02

Accession	Description	Gene Symbol	Fold change	*P*-value
O76094	Signal recognition particle subunit SRP72	SRP72	2.37	1.31E-04
Q14254	Flotillin-2	FLOT2	2.37	2.18E-07
Q15555	Microtubule-associated protein RP/EB family member 2	MAPRE2	2.34	1.47E-02
P35251	Replication factor C subunit 1	RFC1	2.34	4.85E-03
O95232	Luc7-like protein 3	LUC7L3	2.34	3.18E-02
Q9NY12	H/ACA ribonucleoprotein complex subunit 1	GAR1	2.31	1.54E-02
P40222	Alpha-taxilin	TXLNA	2.31	5.40E-04
Q99848	Probable rRNA-processing protein EBP2	EBNA1BP2	2.30	1.60E-03
O95983	Methyl-CpG-binding domain protein 3	MBD3	2.29	9.95E-03
Q71RC2	La-related protein 4	LARP4	2.29	2.79E-06
Q27J81	Inverted formin-2	INF2	2.29	6.28E-06
Q5SYE7	NHS-like protein 1	NHSL1	2.28	4.45E-04
P30825	High affinity cationic amino acid transporter 1	SLC7A1	2.27	7.26E-04
Q03426	Mevalonate kinase	MVK	2.26	3.51E-04
Q96QD8	Sodium-coupled neutral amino acid transporter 2	SLC38A2	2.25	9.02E-06
Q9NRX1	RNA-binding protein PNO1	PNO1	2.25	3.51E-03
Q8NCG7	Diacylglycerol lipase-beta	DAGLB	2.25	3.55E-03
Q12788	Transducin beta-like protein 3	TBL3	2.24	4.57E-03
Q9NR56	Muscleblind-like protein 1	MBNL1	2.23	1.21E-02
P07384	Calpain-1 catalytic subunit	CAPN1	2.22	4.99E-04
Q8WXF1	Paraspeckle component 1	PSPC1	2.22	5.28E-06
Q04323	UBX domain-containing protein 1	UBXN1	2.22	1.58E-03

Accession	Description	Gene Symbol	Fold change	*P*-value
Q9Y6G9	Cytoplasmic dynein 1 light intermediate chain 1	DYNC1LI1	2.21	4.08E-05
Q9GZY8	Mitochondrial fission factor	MFF	2.21	4.11E-04
O60828	Polyglutamine-binding protein 1	PQBP1	2.21	1.23E-02
Q9NUQ6	SPATS2-like protein	SPATS2L	2.21	7.33E-04
Q15286	Ras-related protein Rab-35	RAB35	2.20	7.19E-03
O60547	GDP-mannose 4,6 dehydratase	GMDS	2.20	2.74E-04
Q5T0D9	Tumor protein p63-regulated gene 1-like protein	TPRG1L	2.20	2.79E-04
O00303	Eukaryotic translation initiation factor 3 subunit F	EIF3F	2.20	8.40E-04
P42166	Lamina-associated polypeptide 2, isoform alpha	TMPO	2.19	1.25E-03
P46821	Microtubule-associated protein 1B	MAP1B	2.19	1.44E-05
P45973	Chromobox protein homolog 5	CBX5	2.19	2.30E-04
Q9BVP2	Guanine nucleotide-binding protein-like 3	GNL3	2.18	1.17E-02
P52789	Hexokinase-2	HK2	2.18	1.36E-03
P41567	Eukaryotic translation initiation factor 1	EIF1	2.18	1.19E-04
Q7Z333	Probable helicase senataxin	SETX	2.18	3.11E-04
O00629	Importin subunit alpha-3	KPNA4	2.18	7.50E-06
Q96N67	Dedicator of cytokinesis protein 7	DOCK7	2.17	4.59E-02
Q9NY93	Probable ATP-dependent RNA helicase DDX56	DDX56	2.17	6.91E-03
P20290	Transcription factor BTF3	BTF3	2.17	2.28E-04
Q5JTH9	RRP12-like protein	RRP12	2.16	7.54E-04
P29279	CCN family member 2	CCN2	2.15	5.60E-05
O75683	Surfeit locus protein 6	SURF6	2.15	4.06E-02

Accession	Description	Gene Symbol	Fold change	*P*-value
P23246	Splicing factor, proline- and glutamine-rich	SFPQ	2.14	8.62E-06
Q9H6R4	Nucleolar protein 6	NOL6	2.14	5.26E-05
O14880	Microsomal glutathione S-transferase 3	MGST3	2.13	1.07E-02
Q9Y6M5	Zinc transporter 1	SLC30A1	2.13	1.95E-05
O60271	C-Jun-amino-terminal kinase-interacting protein 4	SPAG9	2.12	3.20E-05
O60701	UDP-glucose 6-dehydrogenase	UGDH	2.12	3.91E-05
P12931	Proto-oncogene tyrosine-protein kinase Src	SRC	2.12	4.18E-03
P46379	Large proline-rich protein BAG6	BAG6	2.12	1.30E-04
Q9Y2W2	WW domain-binding protein 11	WBP11	2.11	9.33E-06
Q6P996	Pyridoxal-dependent decarboxylase domain-containing protein 1	PDXDC1	2.10	1.14E-02
Q9NX58	Cell growth-regulating nucleolar protein	LYAR	2.10	3.67E-03
P69905	Hemoglobin subunit alpha	HBA1; HBA2	2.10	2.81E-06
Q9Y262	Eukaryotic translation initiation factor 3 subunit L	EIF3L	2.10	1.58E-05
Q15424	Scaffold attachment factor B1	SAFB	2.09	3.26E-07
Q14152	Eukaryotic translation initiation factor 3 subunit A	EIF3A	2.09	5.42E-05
P43007	Neutral amino acid transporter A	SLC1A4	2.09	9.59E-07
Q9Y5J6	Mitochondrial import inner membrane translocase subunit Tim10 B	TIMM10B	2.09	4.72E-03
Q96ST2	Protein IWS1 homolog	IWS1	2.09	3.22E-04
Q9P0V9	Septin-10	SEPTIN10	2.08	1.33E-03
Q9Y5K8	V-type proton ATPase subunit D	ATP6V1D	2.08	1.50E-03
Q9H7M9	V-type immunoglobulin domain-containing suppressor of T-cell activation	VSIR	2.08	1.57E-04
Q8WWQ0	PH-interacting protein	PHIP	2.08	4.96E-04

Accession	Description	Gene Symbol	Fold change	*P*-value
Q9BQ61	Telomerase RNA component interacting RNase	TRIR	2.08	3.70E-03
P21283	V-type proton ATPase subunit C 1	ATP6V1C1	2.08	2.21E-05
Q15654	Thyroid receptor-interacting protein 6	TRIP6	2.08	1.28E-04
P78344	Eukaryotic translation initiation factor 4 gamma 2	EIF4G2	2.08	1.28E-03
Q8WTV0-2	Isoform 1 of Scavenger receptor class B member 1	SCARB1	2.07	2.90E-07
Q14203-4	Isoform 4 of Dynactin subunit 1	DCTN1	2.07	6.94E-04
P24534	Elongation factor 1-beta	EEF1B2	2.07	7.33E-06
P42285	Exosome RNA helicase MTR4	MTREX	2.07	3.23E-05
P43490	Nicotinamide phosphoribosyltransferase	NAMPT	2.06	4.01E-03
P11586	C-1-tetrahydrofolate synthase, cytoplasmic	MTHFD1	2.06	5.29E-07
Q86TG7	Retrotransposon-derived protein PEG10	PEG10	2.06	8.84E-07
P60228	Eukaryotic translation initiation factor 3 subunit E	EIF3E	2.06	2.96E-04
A0MZ66	Shootin-1	SHTN1	2.05	2.24E-04
Q14684	Ribosomal RNA processing protein 1 homolog B	RRP1B	2.05	3.07E-02
Q9UNX4	WD repeat-containing protein 3	WDR3	2.05	1.61E-04
Q9Y3Y2	Chromatin target of PRMT1 protein	CHTOP	2.04	1.20E-04
Q9Y315	Deoxyribose-phosphate aldolase	DERA	2.04	1.63E-05
Q15075	Early endosome antigen 1	EEA1	2.03	8.06E-06
O00401	Neural Wiskott-Aldrich syndrome protein	WASL	2.03	7.13E-03
O00159	Unconventional myosin-Ic	MYO1C	2.03	3.85E-04
Q9Y6A5	Transforming acidic coiled-coil-containing protein 3	TACC3	2.03	2.79E-04
Q14847	LIM and SH3 domain protein 1	LASP1	2.03	7.75E-03

Accession	Description	Gene Symbol	Fold change	*P*-value
P17844	Probable ATP-dependent RNA helicase DDX5	DDX5	2.03	3.42E-05
P09601	Heme oxygenase 1	HMOX1	2.03	5.83E-07
Q9BUJ2	Heterogeneous nuclear ribonucleoprotein U-like protein 1	HNRNPUL1	2.03	2.92E-04
Q9NVP1	ATP-dependent RNA helicase DDX18	DDX18	2.02	9.24E-03
Q9NP97	Dynein light chain roadblock-type 1	DYNLRB1	2.01	2.76E-02
P57678	Gem-associated protein 4	GEMIN4	2.01	5.50E-06
O75116	Rho-associated protein kinase 2	ROCK2	2.01	1.88E-05
O75150	E3 ubiquitin-protein ligase BRE1B	RNF40	2.00	1.67E-03
P50579	Methionine aminopeptidase 2	METAP2	2.00	2.54E-02
P11388	DNA topoisomerase 2-alpha	TOP2A	2.00	4.87E-04

(d) Down-regulated proteins comparison KO2 versus WT

Accession	Description	Gene Symbol	Fold change	*P*-value
P07919	Cytochrome b-c1 complex subunit 6, mitochondrial	UQCRH	0.180	2.44E-06
P21953	2-oxoisovalerate dehydrogenase subunit beta, mitochondrial	BCKDHB	0.221	2.32E-03
Q9UQ90	Paraplegin	SPG7	0.230	3.91E-02
Q5VWZ2	Lysophospholipase-like protein 1	LYPLAL1	0.235	3.18E-05
Q92506	(3R)-3-hydroxyacyl-CoA dehydrogenase	HSD17B8	0.239	4.44E-08
Q9BYC8	39S ribosomal protein L32, mitochondrial	MRPL32	0.244	9.69E-03
P50336	Protoporphyrinogen oxidase	PPOX	0.259	1.31E-06
Q15526	Surfeit locus protein 1	SURF1	0.272	3.54E-03
Q9UII2	ATPase inhibitor, mitochondrial	ATP5IF1	0.283	1.37E-05

Accession	Description	Gene Symbol	Fold change	*P*-value
Q8NBL1	Protein O-glucosyltransferase 1	POGLUT1	0.290	3.60E-02
Q9H857-2	Isoform 2 of 5'-nucleotidase domain-containing protein 2	NT5DC2	0.291	3.79E-07
Q12805	EGF-containing fibulin-like extracellular matrix protein 1	EFEMP1	0.306	6.86E-05
Q9NQH7	Xaa-Pro aminopeptidase 3	XPNPEP3	0.309	3.16E-08
P98160	Basement membrane-specific heparan sulfate proteoglycan core protein	HSPG2	0.312	1.27E-05
P12694	2-oxoisovalerate dehydrogenase subunit alpha, mitochondrial	BCKDHA	0.334	1.15E-06
P08697	Alpha-2-antiplasmin	SERPINF2	0.338	1.03E-02
Q9NRK6	ATP-binding cassette sub-family B member 10, mitochondrial	ABCB10	0.343	1.68E-03
O00461	Golgi integral membrane protein 4	GOLIM4	0.346	1.30E-05
Q99523	Sortilin	SORT1	0.360	1.44E-04
Q86SX6	Glutaredoxin-related protein 5, mitochondrial	GLRX5	0.362	5.75E-05
Q14699	Raftlin	RFTN1	0.371	2.98E-03
Q8WWV3	Reticulon-4-interacting protein 1, mitochondrial	RTN4IP1	0.372	3.95E-03
Q99538	Legumain	LGMN	0.374	3.36E-08
Q9Y2U8	Inner nuclear membrane protein Man1	LEMD3	0.381	1.10E-02
Q9H4B0	tRNA N6-adenosine threonylcarbamoyltransferase, mitochondrial	OSGEPL1	0.388	1.27E-05
P18859	ATP synthase-coupling factor 6, mitochondrial	ATP5PF	0.394	2.02E-03
Q8NCN5	Pyruvate dehydrogenase phosphatase regulatory subunit, mitochondrial	PDPR	0.397	5.96E-05
Q8N9F7	Lysophospholipase D GDPD1	GDPD1	0.397	7.58E-03
P43155	Carnitine O-acetyltransferase	CRAT	0.397	2.88E-07
Q96D42	Hepatitis A virus cellular receptor 1	HAVCR1	0.398	3.12E-05
P23434	Glycine cleavage system H protein, mitochondrial	GCSH	0.401	4.07E-04

Accession	Description	Gene Symbol	Fold change	*P*-value
P80303	Nucleobindin-2	NUCB2	0.401	1.10E-05
Q96I59	Probable asparagine--tRNA ligase, mitochondrial	NARS2	0.404	1.50E-03
Q8NFF5	FAD synthase	FLAD1	0.406	5.46E-03
P56589	Peroxisomal biogenesis factor 3	PEX3	0.407	1.04E-04
Q8WVM0	Dimethyladenosine transferase 1, mitochondrial	TFB1M	0.409	2.07E-03
P17301	Integrin alpha-2	ITGA2	0.410	2.05E-02
P04062	Lysosomal acid glucosylceramidase	GBA	0.412	1.41E-07
O95994	Anterior gradient protein 2 homolog	AGR2	0.413	1.05E-04
Q16762	Thiosulfate sulfurtransferase	TST	0.417	1.87E-07
P02787	Serotransferrin	TF	0.419	1.32E-07
O00754	Lysosomal alpha-mannosidase	MAN2B1	0.419	2.92E-05
Q06203	Amidophosphoribosyltransferase	PPAT	0.420	1.20E-03
Q9H2K0	Translation initiation factor IF-3, mitochondrial	MTIF3	0.422	5.09E-05
P09497	Clathrin light chain B	CLTB	0.428	6.12E-05
Q9Y276	Mitochondrial chaperone BCS1	BCS1L	0.435	5.46E-05
P07306	Asialoglycoprotein receptor 1	ASGR1	0.438	1.11E-05
P28300	Protein-lysine 6-oxidase	LOX	0.440	3.78E-05
P13284	Gamma-interferon-inducible lysosomal thiol reductase	IFI30	0.443	1.03E-02
Q92968	Peroxisomal membrane protein PEX13	PEX13	0.449	4.58E-03
P14209	CD99 antigen	CD99	0.450	1.08E-03
Q13740	CD166 antigen	ALCAM	0.451	1.05E-06
Q8NBJ4	Golgi membrane protein 1	GOLM1	0.458	1.46E-06

Accession	Description	Gene Symbol	Fold change	*P*-value
P50416	Carnitine O-palmitoyltransferase 1, liver isoform	CPT1A	0.459	1.04E-07
P22830	Ferrochelatase, mitochondrial	FECH	0.459	7.40E-06
P05166	Propionyl-CoA carboxylase beta chain, mitochondrial	PCCB	0.465	1.46E-05
Q96AX2	Ras-related protein Rab-37	RAB37	0.467	5.18E-05
Q8IVS2	Malonyl-CoA-acyl carrier protein transacylase, mitochondrial	MCAT	0.469	1.18E-02
Q15149-4	Isoform 4 of Plectin	PLEC	0.471	4.03E-05
P04626	Receptor tyrosine-protein kinase erbB-2	ERBB2	0.471	1.05E-04
Q8NF37	Lysophosphatidylcholine acyltransferase 1	LPCAT1	0.473	1.92E-04
P33897	ATP-binding cassette sub-family D member 1	ABCD1	0.480	1.60E-05
Q7L592	Protein arginine methyltransferase NDUFAF7, mitochondrial	NDUFAF7	0.480	1.03E-03
Q8N4Q1	Mitochondrial intermembrane space import and assembly protein 40	CHCHD4	0.484	1.08E-05
Q9NP58	ATP-binding cassette sub-family B member 6	ABCB6	0.488	4.31E-03
P53634	Dipeptidyl peptidase 1	CTSC	0.488	3.45E-06
Q16134	Electron transfer flavoprotein-ubiquinone oxidoreductase, mitochondrial	ETFDH	0.488	1.62E-03
Q9UH99	SUN domain-containing protein 2	SUN2	0.490	2.41E-03
Q9UBR2	Cathepsin Z	CTSZ	0.492	2.12E-06
O15091	Mitochondrial ribonuclease P catalytic subunit	PRORP	0.492	2.43E-04
O00469	Procollagen-lysine,2-oxoglutarate 5-dioxygenase 2	PLOD2	0.493	2.94E-07
P36551	Oxygen-dependent coproporphyrinogen-III oxidase, mitochondrial	CPOX	0.493	1.29E-06
P02751-1	Isoform 1 of Fibronectin	FN1	0.495	1.89E-04
Q9Y5J9	Mitochondrial import inner membrane translocase subunit Tim8 B	TIMM8B	0.500	7.92E-03
Q9BRA2	Thioredoxin domain-containing protein 17	TXNDC17	0.500	2.96E-02

WT, hep G2 cells; KO1, site A and C mutant NPC1 model cells; KO1, site B and D mutant NPC1 model cells.
