# Supplementary figures and images for: Global Proteomics for Identifying the Alteration Pathway of Niemann–Pick Disease Type C Using Hepatic Cell Models
Source: Int J Mol Sci. 2023 Oct 27;24(21):15642. doi: 10.3390/ijms242115642 (PMC10648601; doi:10.3390/ijms242115642)

## Slide 1
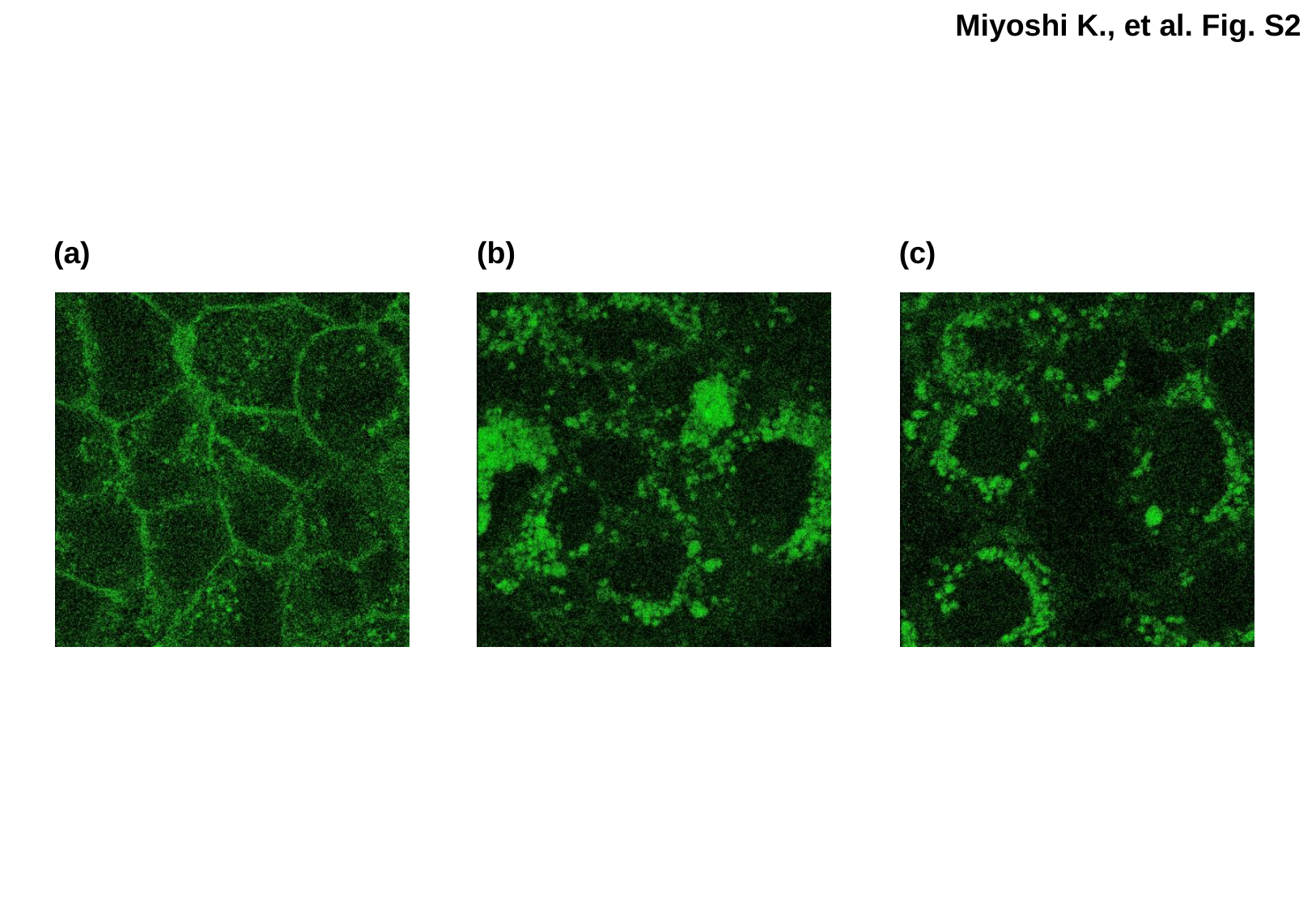

Miyoshi K., et al. Fig. S2
(a)
(b)
(c)

Supplement: Supplementary file 1 [file ijms-24-15642-s001.zip › Figure S2_3.2.pptx]

## Slide 1
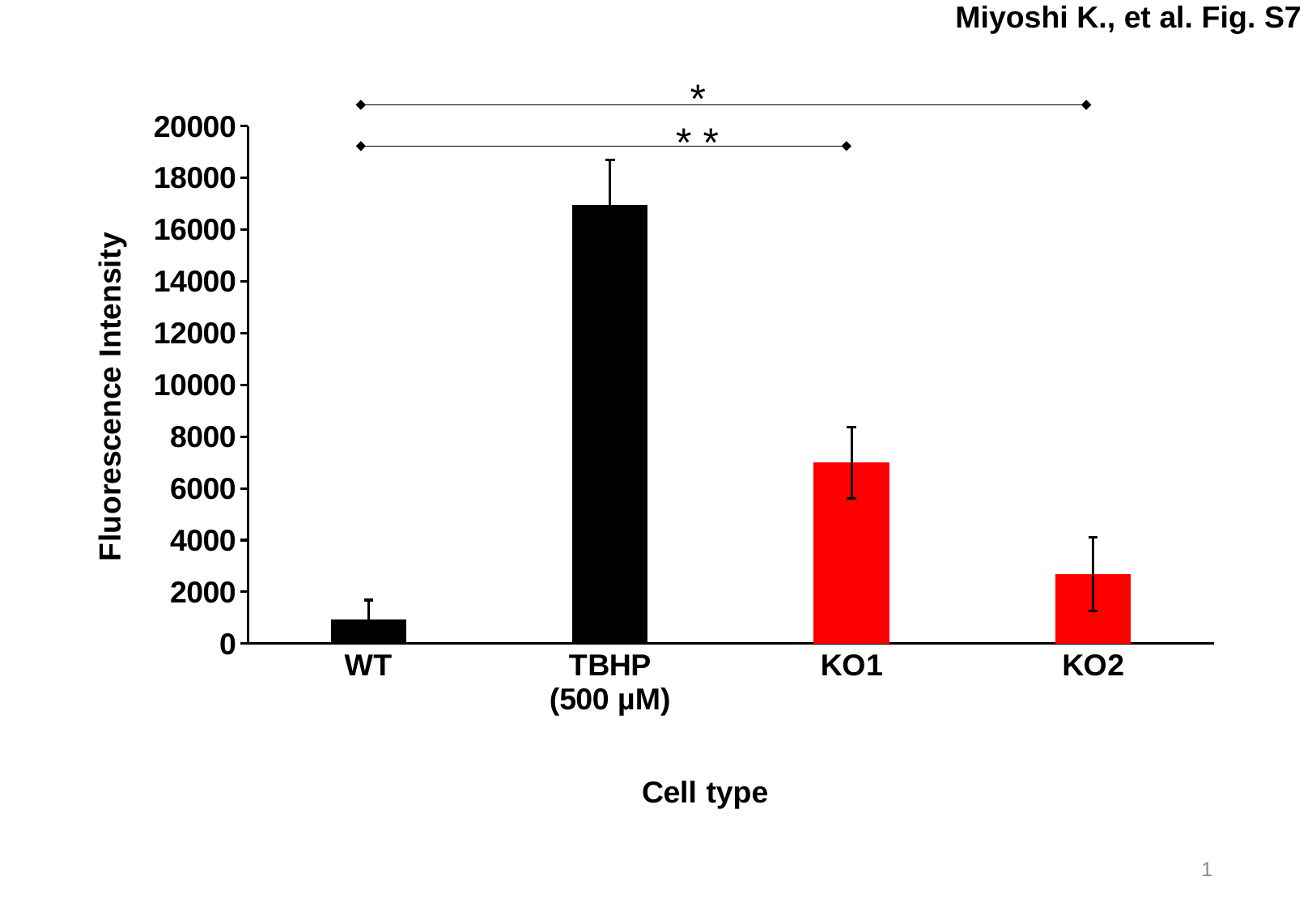

Miyoshi K., et al. Fig. S7
*
### Chart
| Category | |
|---|---|
| WT | 936.8 |
| TBHP
(500 µM) | 16946.0 |
| KO1 | 6994.0 |
| KO2 | 2695.0 |*
*
1

Supplement: Supplementary file 1 [file ijms-24-15642-s001.zip › Figure S7_3.2.pptx]
